# Editorial: Autoimmunity, Infection and Cancer, an Inflammatory Relationship With Intimate Implication to Cancer Prevention and Immunotherapy

**DOI:** 10.3389/fcell.2022.855191

**Published:** 2022-02-11

**Authors:** Zhibin Chen, Richard J. DiPaolo

**Affiliations:** ^1^ Department of Microbiology and Immunology, Miller School of Medicine, University of Miami, Miami, FL, United States; ^2^ Sylvester Comprehensive Cancer Center, Miller School of Medicine, University of Miami, Miami, FL, United States; ^3^ Department of Molecular Microbiology and Immunology, School of Medicine, Saint Louis University, St. Louis, MO, United States

**Keywords:** autoimmunity, infection, cancer, inflammation, senescence, immune therapy

Cancer development in most cases is associated with aging. As such, it was alarming when recent studies uncovered a trend of steady increase of cancer incidence in the young, especially in young females (Anderson et al., 2018; Blaser and Chen, 2018; Kehm et al., 2019). Autoimmunity and infectious agents, with their intertwining inflammatory impact, are suspected to be potential contributors. In animal models, autoimmunity can cause cancer (Nguyen et al., 2013; Miska et al., 2018). A number of infectious agents have long been known for their oncogenic potential (Hatta et al., 2021). This Research Topic, with multi-disciplinary contributions, seeks to examine the molecular and cellular mechanisms underlining autoimmunity, infection and their links to cancer, and the implications of their complex relationship to interventions against autoimmune diseases and cancer.

Among the cancers with increased incidences in the young is gastric cancer. Hoft et al. reviewed two distinct etiological factors of gastric cancer, autoimmunity and *Helicobacter pylori* (Hp) infection. They focused on chronic gastritis, an early condition that can lead to gastric cancer. They outlined the major impact of Hp and the role of autoimmunity in gastritis development, and provided a detailed comparison of Hp-driven gastritis and autoimmune gastritis in clinical manifestation and management, immune profiles and cancer risks.

A common denominator between autoimmunity and infection is inflammation. In a broad “stroke,” inflammation can be “painted” in two categories. In general, type 1 inflammation causes tissue destruction whereas type 2 inflammation leads to damage repair and wound healing. In the review by Li and Chen, they surveyed literature evidence on the association of autoimmunity and cancer, and examined the mechanistic link with a focus on type 2 inflammation. In particular, they delved into how type 2 cytokines IL4 and IL13 might lead to epithelial transformation. Indeed, accumulating evidence suggests that type 2 cytokines may directly affect epithelial differentiation (Miska et al., 2018; Petersen et al., 2018; Noto et al., 2021). At the cellular level, mast cells are perhaps among the most potent innate type 2 immune cells. Noto et al. presented an in-depth review on the role of mast cells in regulating autoimmunity and cancer development through its production of various cytokines, chemokines or vasoactive chemicals. They surveyed evidence for mast cells as a potential mediator between autoimmune diseases and cancer, and illustrated potential targets on mast cells that can be exploited to prevent and treat autoimmune diseases and cancer.

Honan and Chen reviewed another type of cells, the stromal cells, for their potential role in underlining the paths from autoimmunity to cancer development. They discussed the cellular and functional heterogeneity of stromal cells and their involvement in autoimmunity and malignancies, with a mechanistic survey of stromal cell-mediated fibrosis, tissue remodeling, and immune exhaustion or potential cell lineage conversion (Honan et al., 2021). In an established tumor, stromal cells play an integral role in the tumor microenvironment. This is the focus of the review by Ruhland and Alspach. They discussed the stromal cell biology in a context of the paradoxical relationship between senescence and tumors, and examined the impact of direct interactions between senescent stroma and tumor cells (Ruhland et al., 2016) as well as the role of the crosstalk mediated by senescence-associated secretory phenotypes.

The origin of human cancer in most cases is unknown. There are rare cases of germline mutations that cause cancer and those cases can help understand some aspects of human cancer biology. Along that line is the surprising link of human gastric adenocarcinoma with heterozygous CTLA4 mutations. Indeed, in mouse models hypomorphic CTLA4 can cause gastric adenocarcinoma. One of the prominent pathology in this model is the extensive inflammation and metaplasia before transition to frank malignancies (Miska et al., 2018), consistent with a well-accepted paradigm of human gastric tumorigenesis progression from gastritis, metaplasia to adenocarcinoma (Correa, 1988). In this Topic, Sáenz reviewed the phenotypical characteristics of gastric intestinal metaplasia (GIM) and spasmolytic polypeptide-expressing metaplasia (SPEM), and surveyed potential inducers of metaplasia, with a focus on Hp colonization niches (Saenz et al., 2019) and topographic progression of metaplastic lesions. The review examined the clinical implications of metaplasia and potential management strategy of pre-malignancies including risk stratifications and potential prevention of cancer.

Cancer development remains a leading cause of mortality. In the last decade, immune therapy has emerged with unprecedented survival benefits for some advance-staged cancer patients. A fraction of treated patients get years of survival benefit, instead of months of survival with other therapies. However, cancer immunotherapy does incur a substantial burden of immune-related adverse events (irAEs) mediated by autoimmune or inflammatory damage. Mor and Strazza reviewed the potential mechanisms of irAEs induced by immune checkpoint inhibitors (ICIs), focusing on PD-1. They presented an in-depth discussion of PD-1 biology in autoimmunity and cancer, and surveyed the mechanisms and treatment strategy of irAEs. Nevertheless, clinicians often have a “mixed feeling” of irAEs, as those are signs of responses to the ICIs and therefore likelihood of survival benefit. Of note, in animal models, autoimmune T cell clone has been shown to kill tumors (Miska et al., 2012). Regardless how immune therapy works, the biggest challenge is still to get it to work for more patients. It is commonly reasoned that combinatorial therapies are needed and indeed a variety of combinations are tested. Along that line, the review by Zhu et al. could help consider repurposing two drugs, the Bruton’s tyrosine kinase (BTK) inhibitors Ibrutinib and Acalabrutinib. They are effective against B cell malignancies. Zhu et al. present a comprehensive review on their effect on a variety of immune cell subsets besides B cells, and illustrated their impact on BTK-dependent and -independent signaling. Given their broad effect, one can envision that they could be integrated into combinatory regimens to ameliorate autoimmune damage and/or to boost antitumor immunity (Zhu et al., 2021).

We hope this series of articles will help serve as a “primer” or “thought provoker” to understand the complex relationship among autoimmunity, infection and cancer, each presenting a major disease burden to humanity.
